#  Productivity and quality of Dutch hospitals during system reform

**DOI:** 10.1007/s10729-015-9321-7

**Published:** 2015-03-15

**Authors:** Martin van Ineveld, Jeroen van Oostrum, Rob Vermeulen, Adri Steenhoek, Joris van de Klundert

**Affiliations:** 1 Institute of Health Policy and Management, Erasmus University Rotterdam, P.O.Box 1738, Rotterdam, 3000DR The Netherlands; 2 The Health Care Inspectorate, Postbus 2680, 3500 GR Utrecht, The Netherlands

**Keywords:** Hospitals, Productivity, Data envelopment analysis, Malmquist index, Quality indicators, Reimbursement

## Abstract

This study addresses the productivity of Dutch hospitals since the start of the health systems reform in 2005. We consider DEA based measures, which include efficiency and quality for the complete set of Dutch hospitals and present cross-sectional and longitudinal analysis. In particular, we consider how hospital efficiency has developed. As the reform created an environment of regulated competition, we pay special attention to relative efficiency. Our results suggest that the differences in efficiency among hospitals have become larger. In the years 2009–2010, the number of hospitals identified as (close to) efficient by DEA analysis decreased.

## Introduction

At the turn of the millennium, The Netherlands used a closed budgeting system for hospital reimbursement, which was primarily based on hospital wide production parameters. This system was viewed to be supply driven [1] and health insurance companies had little influence on the hospital budget. Under this closed budgeting system, the combination of strict macro budgeting and consistent demand increases caused allocation problems and long waiting lists [2].

The 2005 health system reformed relied on market principles, which created an environment of regulated competition for hospitals [3]. As a result of the reform, health insurers and hospitals freely negotiated prices, volumes and quality for a subset of the health services, which was defined between all stakeholders on a national level. The first year, 2005 can be seen as a transition year in which technological implementation was completed, and public insurance funds disappeared. The freely negotiable services accounted for approximately 10 % of hospital budgets for 2005, 2006 and 2007. This percentage rose to 20 % in 2008 and 33 % in 2009. For most other services the price was set by the National Health Authority (NZa).

The system reform was intended to result in a more demand oriented system in which hospitals delivered higher quality at lower prices [4], i.e., to make hospitals more cost effective [5]. Below we generally refer to the combination of efficiency and quality as performance, and we will define it in detail in the methods section.

The reform not only comprised of a change in reimbursement but also included regulations on public reporting, and supervision of quality by the Health Care Inspectorate IGZ [6]. Some evidence suggests that public reporting is effective as an improvement policy for hospital performance [7]. There is however little evidence regarding the effects of regulated competition in combination with public reporting (or relationship) on hospital performance, or more generally the relationship between these constructs. This holds particularly true for Dutch hospitals, and the recent health reform.

In this study, we explicitly aim to study how the performance of Dutch hospitals has developed since the reform of 2005 which introduced regulated competition and public reporting in The Netherlands. We explore three research questions of which the first addresses overall, average, performance developments, and the remaining two address relative hospital performances:

1. How has the overall performance of Dutch hospitals developed?
2. How have the relative performances developed since 2005?
3. How have less efficient hospitals fared?

The first of this research question addresses the performances of all hospitals and is primarily of a longitudinal nature. The second and third question clearly also have a cross-sectional dimension.

The research can also be viewed to study the health services procurement practices of the insurers. As a result of the reform, the insurers have been given the responsibility to arrange cost reduction and quality improvements for an increasing fraction of the hospital services through their purchasing practices. The study provides insights in the achievements of the insurers. More generally, it is the first DEA study, which presents a longitudinal and cross sectional analysis of hospital performance in The Netherlands in the first 6 years of the reform. The study considers various models to provide robust answers to the research questions formulated above.

## Background

Recent studies for the Netherlands regard various aspects of Dutch hospital performance. Blank et al. [8] explore the productivity of Dutch Hospitals over the period 2003–2009, without explicitly taking the reform into account. They conclude after estimating cost-functions per hospital that productivity has increased by 14,7 % over this 7-year period. They find that scale effects contribute negatively to the productivity growth, and that the relative performance has been stable. They attribute the productivity growth to technological, institutional and societal changes. Ludwig et al. [9] use stochastic frontier analysis on 1998–2002 data – prior to the reform - to study principal agent relationships and efficiency. They conclude that increasing efficiency does not seem to reduce quality, although no relationship was found between the efficiency of departments and the efficiency of the entire hospital. Douven et al. [10] explore the effect of the introduction of managed competition in Dutch inpatient hospital care. Interviewing board members of hospitals, these members suggested the way to raise turnover is to increase production. Bijlsma et al. [11] estimate a bivariate model to consider the relationship between the reform and quality improvement. They find that competition explains differences in performance on process indicators, but not on outcome indicators. Further information regarding health services provided by Dutch hospitals can be found in the yearly reports of The Dutch Healthcare Authorities [12, 13].

Across the Dutch borders, several authors have addressed research questions regarding the relationship between reforms and performance which are akin to the research questions pose above using DEA models. Maniadakis [14] evaluate the first 5 years of the reforms in the UK National Health Service in the early nineties focusing on acute hospitals. Their findings ‘indicate that there was a productivity slowdown in the first year after the reforms but productivity progress in the subsequent years’ resulting in net productivity gain. This gain was mostly attributed to technological change rather than relative efficiency changes. After the first year, they even report a ‘small relative efficiency regress’. Incorporating quality diminished the effects. As is the case for our study, Maniadakis [14] relied on data since the start of the reform.

Other authors have been able to apply a before-after design. Sommersguter-Reichmann [15] evaluates the 1997 Austrian hospital financing reform, using DEA analysis, in particular the Malmquist index. She finds ‘a considerably positive shift in technology between 1996 and 1998 whereas the intended enhancement in technical efficiency has not yet taken place.’ De Castro Lobo [16] consider performance and productivity changes of Brazilian hospitals in the years 2003–2006, in analysis of the 2004 financing reform. Contrary to Sommersguter-Reichmann [15] they find indications that the financing reform provided improvement in the technical efficiency, although the technological frontier has not presented a positive shift.

Finally, Ozcan & Luke [17] studying a reconfiguration of the network of the hospitals of the Veterans Health Administration in the USA. These authors too rely on DEA and Malmquist indices and find indications that the reconfiguration resulted in ‘improvements in overall productivity’ but ‘didn’t produce changes in efficiency’.

## Methods

As the reform has been introduced without distinguishing an intervention group (of hospitals) and a control group (see for instance Hibbard et al. [7] and Lindenauer et al. [18]), and systematic reporting of the required indicators was introduced as part of the reform, we can neither apply a case control design nor a before after design. We therefore rely on a research design where we consider the performance developments over the years 2005–2010, particularly paying attention to performance differences between consecutive years.

Following the references in the background section, we select a standard approach for performance analysis called Data Envelopment Analysis (DEA). DEA is commonly applied in health care, as can be concluded from the review of Hollingsworth [19] who considers 317 efficiency studies, among which 99 are DEA based studies on hospital efficiency (see also Ozcan, [20] and O’ Neill et al. [21]). Since the seminal work on DEA of Charnes et al. [22], it has been repeatedly applied to address the results of hospitals after reform (see for instances Maniadakis et al. [14], Sommersgutter-Reichman [15], De Castro Lobo et al. [16] and Ozcan & Luke [17]).

DEA enables both cross-sectional as well as longitudinal analysis, as befitting our research questions. The longitudinal researchers often use the Malmquist Index (see e.g., [20]), which considers the relative performance improvement between consecutive periods. The Malmquist index has been widely applied to study hospital performance (see e.g., the literature in the background section).

### Data envelopment analysis

DEA formulates efficiency measurement as a linear programming problem. Informally speaking, it solves the problem of measuring performance of each unit in the benchmark set in terms of outputs per inputs, relative to the other units. It explicitly enforces the weighted sum of the outputs to be less than or equal to one (there cannot be more output than input).

1 $$ \frac{{\displaystyle {\sum}_j}{\beta}_j\ {y}_{jk}}{{\displaystyle {\sum}_i}\ {\alpha}_i{x}_{ik}}\kern1em \le \kern0.5em 1\kern1.25em \mathrm{f}\mathrm{o}\mathrm{r}\ \mathrm{all}\ k, $$

2 $$ {\alpha}_i,\ {\beta}_j\ \ge 0\kern1em \mathrm{f}\mathrm{o}\mathrm{r}\ \mathrm{all}\ i,j, $$

Where $$ {\alpha}_i,\;{\beta}_j $$ are the weights of the inputs $$ {x}_{ik}, $$ resp, outputs $$ {y}_{jk} $$, of hospital *k*. The efficiency of hospital *o* is now defined by setting the weights such that the fraction of outputs per input of hospital *o* is maximized:

3 $$ \max \frac{{\displaystyle {\sum}_j}{\beta}_j\ {y}_{jo}}{{\displaystyle {\sum}_i}\ {\alpha}_i{x}_{io}} $$

Thus, the problem of determining the efficiency of a hospital is to select weights which maximize (3), subject to (1) and (2). The set of hospitals for which the objective function attains the maximum value of one define the efficiency frontier which ‘envelops’ the less efficient organizations, whose inefficiency expresses the distance to the frontier [14]. Hospitals on the frontier are often referred to as ‘efficient’.

We now firstly describe the research methods to answer the first research question, and address the methods to answer the other two research questions on this basis.

### Malmquist index

The Malmquist Index (MI) considers the relative performance improvement between consecutive periods as follows. To formally define the Malmquist Index (MI), let ${x}_{ik}^t$ define the values of the inputs in year *t*, and ${y}_{jk}^t$ the values of the outputs in year *t* as before. Let *P(u,v*) be the problem arising when benchmarking the performance of hospital *o* in year *u* against the benchmark set of all hospitals from year *v*. It can formally be defined by taking ${x}_{ik}^u$ and ${y}_{jk}^u$ in the objective function (3) and formulate constraint (1) using ${x}_{ik}^v$ and ${y}_{jk}^v$. Then the Malmquist index *MI* is defined as:

$$ MI=\sqrt{\frac{P\left(t+1,t\right)}{P\left(t,t\right)}\frac{P\left(t+1,t+1\right)}{P\left(t,t+1\right)}} $$

Thus it determines the change in performance of hospital *o* from year *t* to *t + 1* as the geometric means of the benchmark of the year *t* efficiency frontier and the benchmark of the year *t + 1* efficiency frontier. The *MI* is greater than one if and only if the relative performance of hospital *o* has improved from year *t* to year *t + 1*. This Malmquist index serves as our prime measure for the development of hospital performance. As we are interested in the performance development of Dutch hospitals in general, we report for each pair of consecutive years, the Malmquist index averaged over all hospitals.

The MI especially allows providing insight in the development of the efficiency frontier over time, after first rewriting it as follows:

$$ MI=\frac{P\left(t+1,t+1\right)}{P\left(t,t\right)}\sqrt{\frac{P\left(t+1,t\right)}{P\left(t+1,t+1\right)}\frac{P\left(t,t\right)}{P\left(t,t+1\right)}} $$

The first term in this equation is simply the efficiency of hospital *o* in year *t + 1*, divided by its efficiency in year *t*. This relative improvement of hospital *o* against the benchmark is commonly referred to as the efficiency change. The second term expresses how the efficiency frontier has developed taking hospital *o* as a reference and is commonly referred to as the technology change [20]. We explore the performance improvement of efficient hospitals over the years by considering the subsequent average technology changes.

### Choice of DEA model

Before addressing the methods to answers research questions 2 and 3, we first address a number of methodological issues regarding the choice DEA model. The first issue is about the orientation of the DEA model. Input oriented models consider the minimization of inputs for a given level of outputs. Output oriented models maximize the level of outputs for a given level of inputs (See for a further discussion for instance [14, 20]). The same hospitals will appear as efficient under both orientations, but the efficiency scores of the inefficient hospitals are different. As the aim of the reform has been to provide the necessary services efficiently, i.e., to deliver the required output with less input, rather than to increase output, the input oriented model best fits the nature of the reform. This choice is in agreement with the preference for input oriented models from the viewpoint of cost control [21]. Hence, the remainder of the analysis is based on the input oriented model.

A more fundamental issue concerns hospital scale. Constant returns to scale (CRS) models assume a constant rate of substitution between inputs and outputs [20]. In this case, the size of the hospitals is assumed to have no effect on performance. In other words, the productivity function is assumed to be a linear function. Variable returns to scale models (VRS) assume that scale effects take place. Of course, it is common to assume that initially positive returns to scale exist, whereas at some point negative returns to scale may apply. In DEA models, this can be modeled using piecewise linear production functions. As the reform doesn’t consider scale effects but instead aims to improve prices and quality regardless of scale, we firstly consider a CRS model. To explore the sensitivity of our results with regard to scale effects, we also present results for a VRS model.

DEA assumes perfect substitution between inputs and between outputs. Hence, it assumes that an optimal mix of different inputs or outputs can be attained for instance while freely substituting between capital and labor as inputs [23]. Thus, DEA is less suitable if inputs cannot replace each other. Barnum [24] proposes alternative models, which are called fixed factor models. Regarding hospital performance, we recognize that the substitution of technology for labor is possible and hence takes place. At the same time human resources need buildings and equipment to work by and hence these inputs are correlated rather than perfectly substitutable. Our research doesn’t methodologically address the modeling of substitution. However, in order to establish the sensitivity of the results for the choice of model we also present efficiency scores for a fixed proportion ratio model (FPR) [24] and present results on the correlations of the efficiency scores found by the DEA and the FPR models.

### Quality

Historically, DEA has been primarily applied to benchmark efficiency, whereas the health reform not only intended to improve efficiency, but also to increase quality. This quality has been measured in terms of structure, process, and outcome measures [25, 26] by for instance the Dutch Health Inspectorate IGZ. As DEA models disregards the structures and processes by which production takes place, only outcome measures can be included in models, provided that they can be suitably quantified to be incorporated in the outputs. O’ Neill et al. [21] provide examples of the limited number of DEA papers, which have included quality measures. The outcome measures, which are defined hospital wide and collected consistently over the period 2005–2010 are taken into account in the analysis presented below.

### Efficiency classes and market exit

The first research question can be answered using the methods presented above and considering the efficiency frontier and average hospital performances from 2005 to 2010. The second and third research question requires a more detailed analysis of individual hospital performances. DEA methods qualify a hospital as efficient if its efficiency score equals 1. For the purpose of presentation (and without claiming any theoretical interpretation), we have chosen to specify efficiency classes per first digit of the efficiency score. The first class includes the efficient hospitals, the second class includes the hospitals with an efficiency score ranging from 0,9 to 1, a third class with hospitals having efficiency scores from 0,8 to 0,9 et cetera. A hospital can change class from year to year.

To answer the third research question we especially pay attention to hospitals in the lower classes. As low efficiency may indicate high costs levels and/or low quality, a low efficiency score may indicate a less favorable position on the market. Hence, we pay special in the analysis to low scores. This brings about a methodological difficulty however, as we only include hospitals for which we have data for each of the years 2005–2010. Hospitals, which have exited the market are therefore not included, potentially resulting in a biased answer.

We have analyzed the efficiency scores of all hospitals, which have exited the market over the years 2005–2010. In all cases, this has led to a merger or acquisition, and we present efficiency scores before and after the merger whenever possible. As the hospitals involved in mergers or acquisition have by definition not consistently reported efficiency and quality over the years 2005–2010, they are excluded from the DEA benchmark set. Their DEA scores are calculated per hospital by adding it to the benchmark set and solving (1)–(3), however without requiring that their efficiency is bounded by one. By consequence, the efficiency frontier is not affected. The efficiency score of merging hospitals can therefore exceed one. These methods present a small methodological innovation.

## Data and variables

### Data

Study data are obtained from two sources. The first source is ZinData, which has provided financial and production data as available through public reporting. All Dutch hospitals are obliged to report these data in fixed format in their annual reports, which must be approved by accountants. Data are available for the years 2005–2010. The second data source was collected by IGZ and consists of quality measures. The Health Care Inspectorate (IGZ) strives to promote public health through effective enforcement of the quality of health services, prevention measures and medical products. The IGZ advises the responsible ministers and interacts with hospitals by means of advice, encouragement, pressure and coercion. IGZ has the task to ensure that health care providers offer only ‘responsible’ care and to be impartial. http://www.igz.nl/english/ All Dutch hospitals obligatory report quality data to IGZ, which reports the data to the public. We used data that are available for the years 2005–2010 at the public website www.ziekenhuizentransparant.nl.

### DEA input measures

Frequently used input measures for DEA models can be described by three main categories: *capital investment*, *labor*, and *other operating expenses* [21]. More specifically, the following four input measures are the most frequently used: *number of beds*, *operational expenses*, *FTE (full time equivalent) physicians* and *FTE non-physicians*. The measure “number of beds” of Dutch hospital as it is publicly reported refers to the allowed number of clinical beds that a hospital may operate. As this number may deviate substantially from the actual number of beds, and the deviation varies per hospital, we do not include number of beds in our study. Hence, we use three input parameters: FTE employed by the hospital (except physicians), FTE physicians, and the operational expenses.

In contrast to the parameters FTE and FTE physicians, the operating expenses are influenced by inflation. This particularly will influence the longitudinal analysis, as presented through the Malmquist indices. Therefore, the operating expenses for the period 2006–2010 are netted to the price level of 2005. We discount using the official index figures for material costs (without costs for workforce) of the Dutch Healthcare Authority [12, 13]. Since the reform has been initiated, the regulations regarding valuation of assets have been repeatedly and significantly adjusted for Dutch hospitals over the period 2005–2010. These adjustments were made to account for changes in the reimbursement system for capital costs, after which capital costs have ceased to be reimbursed separately and the Dutch state is no longer involved in carrying risks of capital costs. By consequence, the mandatory reported balance sheet figures for fixed assets over the years 2005–2010 are inconsistent and therefore unfit to be considered as an input variable in the DEA models. On average, capital costs account for less than 10 % of total costs [27].

### DEA output measures

To be congruent with important parameters in the current Dutch reimbursement system for hospitals and with many other DEA studies (see e.g., [19] we consider as output measures: *number of (inpatient) admissions*, *number of primary outpatient visits* and *day care treatment*. These numbers have been a primary basis for reimbursement before 2005 and have remained important afterwards.

### Quality measures

Quality measures are adopted relatively recently compared to financial and production-oriented measures. This has implications for the quality of the data supplied to the Dutch Ministry of Health. Moreover, many definitions of quality indicators have been adapted over time and many quality indicators have disappeared or are replaced by others over the years 2005–2010. The latter especially occurred when indicators are no longer discriminating among hospitals. As a result, very few outcome indicators have been consistently collected over the period of analysis 2005–2010.

We have selected quality measures based upon the following criteria:

- Available in all years (2005–2010)
- Indicator is measured correctly and uniformly within hospitals and over the years considered
- The indicator addresses the hospital as a whole (as opposed to a single unit or medical discipline).

Table 1 shows all IGZ quality measures and their appropriateness for inclusion. Given the impact of the system change and the aim to improve quality it is remarkable that only two hospital-wide quality measures have been consistently collected since 2005: Prevalence of decubitus ulcers and cancelled surgical operations (except cancellations by patients). Both these quality indicators are ratios: the percentage of cancelled elective surgeries and the percentage of inpatients having decubitus. Ratios as such cannot function as an input/output parameter in DEA [28]. A feasible alternative for both ratios is to correct the output volumes, by only counting as production the volume of surgeries that have not been cancelled, or the volume of admissions, which have not developed decubitus ulcers. As surgery volume is not an output variable in our model, we cannot correct for surgery cancellation, and thus further disregarded this parameter. Hence, decubitus ulcers prevalence is the only quality measure taken into account in our DEA analysis. When taking it into account, we reduce the inpatient admission by the fraction of patients for which decubitus ulcers have been reported.

**Table 1:** IGZ quality indicators selected scoring positive on the three mentioned criteria

	Are data available over the period 2005–2010 and have relevant definitions remain consistent?	Is it an outcome measure?	Is it a hospital wide measure?	Inclusion (‘yes’ in each of the previous columns)
Cancelled surgeries	Yes	Yes	Yes	Yes
Stroke	No	Yes	No	No
Decubitus ulcers	Yes	Yes	Yes	Yes
Hip fracture	Yes	Yes	No	No
Intensive Care	Yes	No	Yes	No
Breast cancer	No	Yes	No	No
Pain after surgery	Yes	Yes	No	No
AMI	Yes	Yes	No	No
Blood transfusion	No	Yes	Yes	No
Cataract	Yes	No	No	No
Cholecystectomy	Yes	Yes	No	No
Complication registration	Yes	No	Yes	No
Diabetes	Yes	Yes	No	No
Heart failure	Yes	Yes	No	No
Pediatric surgery	No	Yes	No	No
Medication safety	No	No	No	No
Malnutrition	No	Yes	Yes	No
Unplanned reoperation	No	Yes	No	No
Post-operative wound Infection	No	No	Yes	No
High risk interventions	No	No	No	No
Hospital infections	No	No	Yes	No
ICT infrastructure	No	No	Yes	No
Pregnancy	No	Yes	No	No
Hospital standardized mortality rate	No	Yes	Yes	No
Unexpected extended length of stay	No	Yes	Yes	No

### Exclusion of hospitals

University hospitals have been excluded from the study as they form joint organizations with the medical faculties and therefore have differences in input variables with the general hospitals. Moreover, their objectives explicitly include research and education and therefore consider different outcome variables as well. Finally, academic hospitals function as tertiary hospitals and have a case-mix with a considerably higher complexity. Specialty hospitals have also been excluded.

Since we aim to study efficiency trends over 6 years, we have excluded hospitals that do not report the mandatory data in all years. Table 2 presents a descriptive overview of all measures for the selected hospitals. As mentioned in the methods section, however, a separate analysis is performed on excluded hospitals that have merged or been acquired.

**Table 2:** Descriptive statistics for DEA variables for the included Dutch hospitals (*n* = 65) over 2005–2010

Variable	Mean	SD	Minimum	Maximum
Input				
FTE (except physicians)	1520	807	408	4186
FTE Physicians	114	52	33	255
Operating expenses	€43.158.768	€25.705.833	€10.468.000	€131.845.207
Output				
Number of admissions	19.673	8582	6141	47.040
Number of day care treatments	18.844	9178	4702	52.411
First outpatient visits	114.197	47.258	37.574	255.948
Quality (*n* = 59)				
Percentage decubitus	4,3	2,4	0,0	15,1
Percentage decubitus	4,3	2,4	0,0	15,1

## Results

We present the results following the sequence of analysis by different models which firstly regard productivity. We then include the sole quality parameter meeting the inclusion criteria. We conclude by considering the efficiency of hospitals which have merged or been acquired.

### DEA constant returns to scale

Table 3 shows the DEA results per year for an input-oriented constant return to scale model for the years 2005–2010 in which quality is not taken into account. The average efficiency is consistently at or slightly below 0,9 for 2005–2008, and then declines to 0,87 in 2009 and 0,85 in 2010.

**Table 3:** DEA results with economic parameters only in an input-oriented CRS model for Dutch hospitals (*n* = 65)

Efficiency level	2005	2006	2007	2008	2009	2010
1,0	15	15	12	12	13	9
≥0,9–<1,0	15	14	17	22	15	15
≥0,8–<0,9	28	24	27	24	21	18
≥0,7–<0,8	5	10	7	6	11	18
≥0,6–<0,7	2	2	2	1	5	5
≥0,5–<0,6	0	0	0	0	0	0
Average efficiency	0,90	0,89	0,89	0,90	0,87	0,85
SD efficiency	0,09	0,09	0,09	0,08	0,10	0,11

In the input oriented CRS DEA model results presented in Table 3, the number of efficient hospitals declines from 15 in 2005 to 9 in 2010. At the same time we notice that the number of hospitals with relative efficiencies below 0,8 increase from 7 in 2005 to 23 in 2010.

Table 4 gives the Malmquist Indices for the year-to-year development of efficiency. It shows small overall improvements, except from 2009 until 2010. Notice that values of greater than one for technological change indicate a small but consistent development of the increased efficiency at the frontier over the years. It is also worth considering the average efficiency changes, which reflect the average distance to the efficiency frontier. Table 4 confirms the finding of Table 3 that the average relative efficiency decreases over the years 2009, 2010. In 2009, this is accompanied by an increased technological change, which is not the case for 2010.

**Table 4:** Malmquist Index with economic parameters only in an input-oriented CRS model for Dutch hospitals (*n* = 65)

	2005–2006	2006–2007	2007–2008	2008–2009	2009–2010
Malmquist index	1,01	1,02	1,02	1,02	0,99
Efficiency change	0,99	1,00	1,01	0,96	0,97
Technology change	1,03	1,01	1,01	1,05	1,02
Malmquist index standard deviation	0,05	0,05	0,07	0,06	0,07
Malmquist index – minimum value	0,87	0,83	0,75	0,75	0,87
Malmquist index – maximum value	1,13	1,11	1,15	1,08	1,36
Malmquist index – maximum value	1,13	1,11	1,15	1,08	1,36

Regarding research question 3, Table 5 presents results on the reoccurrence of low efficiency scores over the years. It shows that 10 of the 65 hospitals have efficiency score of 0,8 or less in at least 4 of the years 2005–2010, while this is the case for 4 hospitals in each of the reported years. These hospitals account for around three quarters of the efficiency scores less than 0,8 between 2005 and 2009, and form almost half of the 23 hospitals with efficiency less than 0,8 in 2010. The majority of hospitals always had efficiency above 0,8. The recurrence frequency of having a score below 0,8 in subsequent years is 87 %.

**Table 5:** Frequencies of low efficiency scores during the period 2005–2010 (*n* = 65)

Frequency of scores below 0.80	Number of hospitals
0	38
1	11
2	6
3	0
4	3
5	3
6	4

### DEA variable returns to scale

Table 6 presents the DEA results when using a VRS model instead of a CRS model. One notices that the result displays the same trend, yet that the average efficiency scores are now about 0,05 higher. In particular, we notice that the number of efficient hospitals has approximately doubled and is now more stable until 2010. The number of hospitals with efficiency scores of at most 0,8 is quite stable over the period 2005–2010. When making this comparison, however, let it be noted that the bounds of 0,9; 0,8 et cetera have not been adjusted for changing the model.

**Table 6:** DEA results with only economical parameters in an input-oriented VRS model for Dutch hospitals (*n* = 65)

Efficiency level	2005	2006	2007	2008	2009	2010
1,0	27	26	29	28	29	21
≥0,9–<1,0	19	23	19	24	21	23
≥0,8–<0,9	17	14	16	11	11	17
≥0,7–<0,8	2	2	1	2	4	4
≥0,6–<0,7	0	0	0	0	0	0
≥0,5–<0,6	0	0	0	0	0	0
Average efficiency	0,94	0,94	0,94	0,95	0,95	0,93
SD efficiency	0,07	0,07	0,07	0,06	0,07	0,07

**Fig. 1:**
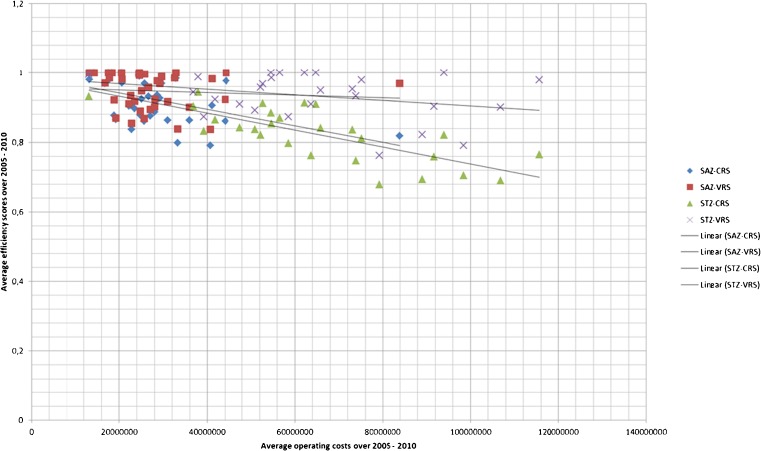
DEA scores versus average total operating expense over the years 2005–2010

Figure 1 presents linear regression plots of average efficiency versus average total operating expenses over the years 2005–2010 for VRS and for CRS efficiency scores. It distinguishes the non-academic but ‘top clinical’ STZ hospitals from the other ‘general’ hospitals (named SAZ in Fig. 1), as STZ hospitals may have a different case mix. Figure 1 clearly displays a scale effect by showing that larger hospitals are less efficient by the negative correlation between VRS score and scale and by the increasing differences in VRS and CRS scores as a function of scale. Any case mix differences, which may exist between STZ and non STZ hospitals, however, do not translate to differences in efficiency between them.

### Fixed proportion rate

Table 7 presents the results when using a FPR model. As the model doesn’t allow scale effects or substitution between inputs, the average efficiency scores are lower than in the CRS and VRS modeled presented above. We have calculated paired samples statistics of the CRS and the FPR models and found that they are highly (0,896) and significantly correlated. By definition, there is only one efficient hospital in this analysis. Likewise, we notice that the number of hospitals with a score of 0,9 or higher has decreased. Like the VRS model, The FPR model finds that the average relative efficiency decreases and that in 2010 there are more hospitals with low efficiency scores.

**Table 7:** Fixed Proportion Ratio results with only economical parameters for Dutch hospitals (*n* = 65)

Efficiency level	2005	2006	2007	2008	2009	2010
1,0	1	1	1	1	1	1
≥0,9–<1,0	13	10	7	13	10	4
≥0,8–<0,9	16	19	18	29	25	20
≥0,7–<0,8	26	22	28	15	18	25
≥0,6–<0,7	5	9	8	5	9	11
≥0,5–<0,6	4	4	3	2	2	4
Average efficiency	0,80	0,79	0,79	0,82	0,81	0,76
SD efficiency	0,10	0,11	0,10	0,10	0,10	0,10

### Quality

Table 8 shows the results of incorporating decubitus ulcers as a means of quality adjusted for the patient admissions output parameter in the CRS-DEA model. The paired samples statistics of the CRS with and without the adjustment for decubitus are very close to 1 (0,989) and significant. Notice that the total set of hospitals is not 65 but 59, as not all hospitals have reported the quality data consistently over the years 2005–2010. We observe that the number of efficient hospitals is initially slightly higher yet decreases more than in the corresponding model which doesn’t adjust for decubitus. Likewise, the number of hospitals with scores of 0,9 or higher decreases more sharply. The number of hospitals with scores of at most 0.8 now increases from 8 in 2005 to 22 in 2010. The average relative efficiency decreases sharper than in the model with adjustment.

**Table 8:** DEA results where admissions have been adjusted for decubitus ulcers in an input-oriented CRS model for Dutch hospitals (*n* = 59)

Efficiency level	2005	2006	2007	2008	2009	2010
1,0	16	15	12	10	11	7
≥0,9–<1,0	15	12	18	20	13	12
≥0,8–<0,9	20	23	22	21	19	18
≥0,7–<0,8	6	7	5	7	11	18
≥0,6–<0,7	2	2	2	1	5	4
≥0,5–<0,6	0	0	0	0	0	0
Average efficiency	0,90	0,90	0,89	0,90	0,86	0,84
SD efficiency	0,09	0,09	0,09	0,09	0,10	0,10

### Merged hospitals

Finally, we present the results required to the answer the second part of research question 3, by considering the efficiency scores of hospitals which have exited the regulated market during the period of analysis 2005–2010. Recalling that each of these hospitals has merged or was acquired, Table 9 presents analysis for the corresponding pairs of hospitals. Notice that as discussed in the Methods section, efficiency scores exceeding one do indeed occur. Hospital 6 was included in the initial benchmark set. It is presented below because it acquired Hospital-6A, however with very little impact on efficiency.

**Table 9:** DEA results for excluded hospitals, either merged or acquired, (CRS), excluded hospitals are benchmarked against included

		2005	2006	2007	2008	2009	2010
Merge	Hospital-1 A	0,8333	0,8170	0,8296	0,7794		
	Hospital-1 B	0,7749	0,7697	0,8565	0,9143		
	Hospital-1 A+B					0,7384	0,7090
Merge	Hospital-2 A	na					
	Hospital-2 B	na					
	Hospital-2 A+B		0,8282	0,8220	0,7585	0,6740	0,6258
Merge	Hospital-3 A	1,0299	1,0939	1,1963	1,0670		
	Hospital-3 B	0,8262	0,7231	0,7643	0,7853		
	Hospital-3 A+B					0,5541	0,7682
Merge	Hospital-4 A	1,1084	1,0211	1,0064	na		
	Hospital-4 B	1,0000	0,9484	1,1579	na		
	Hospital-4 A+B				na	1,0312	0,8727
Merge	Hospital-5 A	na					
	Hospital-5 B	na					
	Hospital-5 A+B		na	na	na	0,7512	Na
Acquisition	Hospital-6A	na	na	na			
	Hospital-6	0,6243	0,6359	0,6582	0,6766	0,6828	0,6359

*na* = not available

We notice that of the hospitals which have merged only hospital 3-B reported an efficiency of less than 0,8 for more than 1 year prior to merger. Unfortunately, data for hospitals 2A, 2B, 5A, 5B, and 6A are missing for the years prior to merger/take-over. We notice that in the 3 cases for which data was available, the efficiency scores after the merger are lower than the unweight average of the efficiency score before the merger., In 2 out of 3 cases, the efficiency score after the merger is less than the minimum score between the two hospitals prior to merger. The efficiency of the other 3 pairs is less than 0.8 after merger.

## Conclusion

Lacking control group data, we cannot attribute any (absence of) development in performance to be an effect of the reform. Although 2005 can be viewed as a baseline, there is no data which is truly from before the reform. Hence, before-after analysis regarding before versus after reform performance differences can neither be made. Our conclusions are simply based on the various efficiency developments as observed after the reform was introduced in 2005.

Regarding the first research question, we observe from Table 3 that, until 2008, the relative average performance has been stable at 0,90 while – as shown in Table 4 - the technological efficiency slightly increased as the technological efficiency shows small improvements of 1 or 2 %. For the year 2009, the Malmquist analysis reveals a higher increase in the technological change to 5 %, which drops back to a more usual 2 % in 2010. 2010 is however the first year where the Malmquist index doesn’t show a positive average plus of around 2 % improvement, but a small negative average improvement.

To answer the second research question, the DEA analysis presented in Table 3 shows that the relative average performance decreased in 2009 and 2010 to 0,87 and 0,85 respectively. As this average decrease has been accompanied by an increase in technological change in 2009, the increase in technological improvement by the efficient hospitals explains most of the larger efficiency differences. For 2010, the larger differences are however not explained by increased technological improvement, and can therefore be considered to be caused by decreases in efficiency of non-efficient hospitals. The increased standard deviations of the efficiency scores in 2009 and 2010 confirm these conclusions.

These findings seem partly influenced by the choice of model. The VRS model results display a more stable efficiency development from 2005 to 2010. Average and standard deviation of the efficiency scores remain by and large unchanged, perhaps with a small decline in efficiency in 2010. In combination with the results presented in Fig. 1, the CRS and VRS results suggest that the efficiency gap between smaller and larger hospital has grown larger in 2009 and 2010, and that the VRS parameters compensated for this effect.

The FPR model provides results, which are comparable to the DEA models. The FPR model displays a stable average efficiency of around 0,8 which drops to 0,76 in 2010 while the variance is stable around 0,1 throughout. This might indicate that from a fixed proportion viewpoint, especially the most efficient hospital improved in 2010, while the developments have otherwise been quite stable.

Likewise, inclusion of the sole quality outcome parameter that met the inclusion criteria (it regards decubitus ulcer prevalence) had only a marginal impact on the CRS results. The results don’t provide evidence that inclusion of quality measures leads to added insights on performance developments. However, this is mainly due to the definition and stability of the available quality indicators.

With regard to the third research question, we find that there are around 10 hospitals, which have consistently been at least 20 % less efficient than the most efficient ones for the majority of years in a 6 years period. Given the choice of model and input variables this means that they have employed 20 % more physicians and/or staff and/or incurred 20 % higher, other operating costs for the same level of output than efficient counterparts. These 10 hospitals account for around three quarter of such inefficiency until 2009. The conclusions above indicate that several larger hospitals have joined these less efficient performers in the years 2009 and 2010, after the fraction of freely negotiable services increased to 33 % in 2008.

Our analysis provides no evidence that merger (take-over) was related to lack of efficiency before the merger (take-over): the average efficiency scores of the hospitals that have merged have been between 0,89 and 0,96 over the years 2005–2008. Conversely: the average efficiency scores after the merger or acquisition have been between 0,62 and 0,74, placing the resulting hospitals on average among the worst performing hospitals from the benchmark set. The argument of selection bias, which was a prime motivation to consider the merged hospitals thus worked counter to our expectations.

## Discussion

Before, providing a more general reflection, let us discuss the methodological strengths and weaknesses of the presented analysis and its consequences. As mentioned, the study design inhibits attribution of effects to the reform. Nevertheless, we can observe that the average efficiency of the hospitals has been stable around 0,90 (CRS) or 0,95 (VRS) for the first 3 years after the reform in which the fraction of health services for which prices have been freely negotiable was around 10 %. In 2008, resp. 2009, this fraction increased to 20 %, resp. 33 %, while the CRS average efficiency change dropped from being very close to 1.00 to 0,96 and 0,97 respectively while the technological change increased to 1,05 and 1,02. Our analysis has not revealed a consistent relation between increases in market share of freely negotiable health services and performance change in the same year. Despite the methodological limitations, the 2009 and 2010 technology changes reported in Table 4, in combination with Fig. 1, suggest the following. The reform expanded the fraction for which prices and volumes were freely negotiable. Some of the smaller hospitals managed to achieve efficiency improvements intended by the reform, whereas the efficiency changes reveal that many larger hospitals typically did not.

No model will perfectly capture the efficiency and quality developments of Dutch hospitals since 2005. Therefore, we modeled these developments using a variety of models, and although there is some variation in the results, they confirm rather than contradict each other. Moreover, combining the VRS with the CRS results leads to additional insights. We also notice that the results of the axiomatically different FPR model are highly correlated with the CRS model results. In addition, we observe that the efficiency scores obtained by including quality data are very closely correlated to the initial CRS model efficiency scores.

Considering that the FPR model results displays the same delay in the performance decrease as the VRS model, one may suggest that the scale and substitution effects are interrelated. Have smaller, efficient hospitals started to realize substitutions in 2009, which larger hospitals didn’t realize?

As the model which includes quality by and large provides the same results as the CRS model, it supports the viewpoint that quality improvement and economic efficiency go hand in hand (and doesn’t conform the viewpoint that more money yields higher quality). Overall, the model triangulation in the research design has strengthened and enriched the findings.

The results are based on using FTEs and operating costs as inputs, while capital is not considered as an input due to inconsistency of capital valuation regulations over the study period. Although capital costs constitute less than 10 % of total costs [27], inclusion of capital as a production factor is worthy of future research. Regarding the quality, it has been somewhat disappointing that only two outcome related quality indicators have been consistently and reliably collected over the years 2005–2010. In particular, since improving quality has been an explicit objective of the reform. This objective therefore appears to be difficult to evaluate for quality at the hospital level.

The set of included hospitals is as nationwide and complete as public reporting allows and considers 6 consecutive years since the reform has been implemented in 2005. Some bias may exist, as not all hospitals are included. Some are excluded because they are academic hospitals or specialty hospitals, others because they have not consistently provided the legally due information. We have explicitly addressed hospitals not selected in the benchmark because of mergers/take-overs.

Although the original closed budgeting system by which at least two third of the average hospital budget was reimbursed over the years 2005–2010 weighs production for different medical specialties differently, case mix is not explicitly accounted for in our study. One may argue that case mix differences exist as some hospitals provide a more extensive service product mix and/or treat more complex patients. The top clinical hospitals (STZ) distinguish themselves from the remaining hospitals by providing a broader product mix and serving more complex patients. Figure 1 however doesn’t confirm differences in case mix between STZ hospitals and other hospitals to explain efficiency differences. On the other hand, we cannot rule out a relationship between theses case mix determinants and diminishing scale effects. More generally, we encourage further research explaining the relationship between scale and case mix of Dutch hospitals. This topic is relevant in the ongoing debate regarding the optimal numbers of hospitals and patient volumes in The Netherlands.

The case-mix may not only vary across the hospitals, but also over the years. The population has changed as life expectancy has increased over the years 2005–2010. Moreover, parallel reforms took place in primary care, resulting in a shift in treatment for chronic diseases such as Type 2 Diabetes. Thus, over the study period 2005–2010 a substitution took place from hospital care to primary. Similarly, there have been innovations leading to substitution of care from the tertiary academic hospitals to the general hospitals, and new more effective treatments practices have been adopted by the hospitals otherwise. The performance developments as reported in our study don’t include or correct for these contextual changes and technological advancements that the selected hospitals have dealt with over the years 2005–2010.

The average input-oriented CRS DEA scores of around or slightly below 0,9 are slightly higher than the average (CRS and VRS) scores found for EU hospitals of 0,86 by Hollingsworth [19], but slightly below the average of 0,91 reported by O Neill et al. [21]. The average input-oriented VRS DEA score are considerably higher than both these scores, despite the fact that the models use only 3 inputs and 3 outputs. This suggests that the average efficiency of Dutch hospitals (when benchmarked against each other) over the benchmark period is quite high in comparison to other European countries (when benchmarked within their respective countries).

Although the research design inhibits attribution of the efficiency developments to the reform, the reduced average efficiency in the last 2 years are worthy to consider in relation to the reform. On the one hand, one may argue that - counter to the intention of the reform – hospitals which experience less competition, e.g., for geographical reasons, became less efficient when the regulated competition allowed them. An alternative viewpoint is that insurers continued the purchasing practices, which were in place before the reform as even after the reform was introduced prices and volumes were not freely negotiated for the vast majority of services. Likewise hospital management practices may have continued to be primarily aligned with the system by which the majority of services continued to be reimbursed. Therefore, the reform may not have had a substantial impact on hospital efficiency until 2010, but rather initiated purchasing, management and reporting changes as necessary for progression of the reform. Progress is taking place as the fraction of services for which prices and volumes can be freely negotiated has increased to 70 % by 2014 and insurers have implemented more selective purchasing practices in recent years. Research regarding the years 2011 onwards can shed further light on the developments as the reform progresses. Such further research may also address the aforementioned geographical determinants of competition, as well as differences in purchasing and management practices.
